# Redução dos Níveis de Insulina em Pacientes com Doença de Chagas Aguda na Amazônia Brasileira Tratados com Benznidazol

**DOI:** 10.36660/abc.20240621

**Published:** 2025-05-08

**Authors:** João Marcos Barbosa-Ferreira, Jessica Vanina Ortiz, Fernanda Gallinaro Pessoa, Maria das Graças Vale Barbosa, Jorge Augusto de Oliveira Guerra, Kátia do Nascimento Couceiro, Mônica Regina Hosannah Silva e Silva, Matheus Martins Monteiro, Keila Cardoso Barbosa Fonseca, Felix Alvarez Ramires, Barbara Maria Ianni, Charles Mady, Fábio Fernandes

**Affiliations:** 1 Universidade do Estado do Amazonas Manaus AM Brasil Universidade do Estado do Amazonas, Manaus, AM – Brasil; 2 Programa de Pós-Graduação em Medicina Tropical Universidade do Estado do Amazonas Manaus AM Brasil Programa de Pós-Graduação em Medicina Tropical – Universidade do Estado do Amazonas, Manaus, AM – Brasil; 3 Hospital das Clínicas Faculdade de Medicina Universidade de São Paulo São Paulo SP Brasil Instituto do Coração do Hospital das Clínicas da Faculdade de Medicina da Universidade de São Paulo, São Paulo, SP – Brasil

**Keywords:** Insulina, Adipocinas, Doença de Chagas, Ecossistema Amazônico, Cardiomiopatia Chagásica

## Abstract

**Fundamento:**

Na região Amazônica, observou-se um aumento considerável no número de casos de doença de Chagas aguda (DCA), resultando em alterações eletrocardiográficas e ecocardiográficas. Os principais mecanismos fisiopatológicos da cardiomiopatia chagásica (CC) incluem a disfunção microvascular, a disfunção do Sistema Nervoso Autônomo (SNA), a agressão direta do parasita e a atividade inflamatória. A doença de Chagas (DC) é um ótimo exemplo de cardiomiopatia inflamatória, capaz de influenciar alterações nos parâmetros metabólicos e no SNA. Há uma escassez de estudos que abordam a medição dos níveis de adipocitocina e insulina em indivíduos com DCA.

**Objetivo:**

Avaliar os níveis de adiponectina, leptina e insulina antes e após o tratamento da DCA com benznidazol e estabelecer correlações com o SNA e a função cardíaca.

**Métodos:**

Foram incluídos 28 indivíduos, os quais foram divididos em: grupo controle (GC), com 15 indivíduos, e grupo com DCA, com 13 indivíduos. Todos os indivíduos foram submetidos a um ECG padrão de 12 derivações, ecocardiografia transtorácica completa, avaliação da função autonômica e dos níveis séricos de adiponectina, insulina e leptina. Foi adotado um nível de significância de 5% (valor de p < 0,05).

**Resultados:**

Os níveis de insulina foram mais baixos no grupo com DCA em comparação com o grupo controle, tanto antes quanto após o tratamento, e apresentaram redução na fase pós-tratamento em comparação com a fase pré-tratamento. Não houve diferença nos níveis de adipocitocinas, leptina e adiponectina entre os grupos.

**Conclusão:**

Os níveis de insulina foram mais baixos no grupo com DCA em comparação com o grupo controle, tanto antes quanto após o tratamento, e apresentaram redução na fase pós-tratamento em comparação com a fase pré-tratamento. Os níveis de adipocitocinas e insulina não apresentaram correlação significativa com os parâmetros de função cardíaca e do SNA.

## Graphical abstract

**Figura Central:**
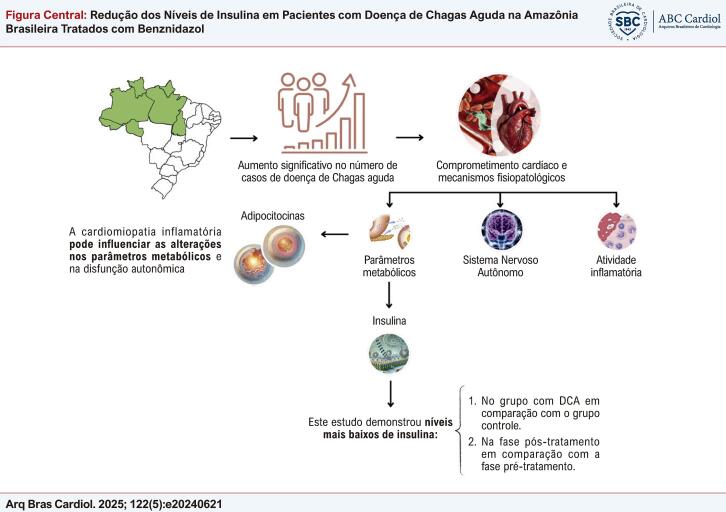
Redução dos Níveis de Insulina em Pacientes com Doença de Chagas Aguda na Amazônia Brasileira Tratados com Benznidazol

## Introdução

A doença de Chagas (DC) está presente desde o sul dos Estados Unidos até a Patagônia e afeta cerca de 8 milhões de pessoas na América Latina.^1^ Além disso, devido ao aumento do fluxo migratório, a DC está se tornando cada vez mais relevante em países não endêmicos, como os Estados Unidos, o Canadá, alguns países da Europa, o Japão e a Austrália. Estima-se que, nos Estados Unidos, a doença pode ter infectado 300 mil imigrantes legais. A Espanha possui a segunda maior prevalência da doença, com cerca de 40 a 60 mil imigrantes infectados.^2^ No Brasil, mais precisamente na região Amazônica, houve um aumento significativo no número de casos nas últimas décadas, principalmente na fase aguda da doença, associada ao consumo de sucos de frutas regionais contaminados. Foram relatados casos de comprometimento cardíaco, além de alterações eletrocardiográficas e ecocardiográficas.^3^

A evolução para disfunção miocárdica representa a principal causa de morbidade e mortalidade na DC, afetando cerca de 30% dos pacientes. Os principais mecanismos fisiopatológicos da cardiomiopatia chagásica (CC) incluem: disfunção microvascular, comprometimento do Sistema Nervoso Autônomo (SNA), agressão direta do parasita e atividade inflamatória grave.^4^ Alguns desses mecanismos também têm o potencial de influenciar alterações nos parâmetros metabólicos em pacientes com DC, como a disfunção autonômica e a atividade inflamatória. Vários dos principais mediadores da atividade metabólica são secretados pelo tecido adiposo (adipocitocinas) ou fazem parte do metabolismo da glicose.

As adipocitocinas são mediadores bioativos produzidos pelo tecido adiposo. As principais adipocitocinas são a adiponectina e a leptina. A adiponectina possui efeitos anti-inflamatórios e antiaterogênicos benéficos, além de ação de sensibilização à insulina.^5 - 9^ Entretanto, o papel da adiponectina nas doenças cardiovasculares ainda é controverso. Estudos anteriores demonstraram que os níveis de adiponectina são elevados em pacientes com insuficiência cardíaca (IC) sistólica, prevendo até mesmo morbidade e mortalidade.^10 , 11^ A leptina estimula atividades pró-inflamatórias e pró-trombóticas, proliferação neointimal, disfunção endotelial e indução de resistência à insulina.^12^

O *Trypanosoma cruzi* apresenta tropismo pelo tecido adiposo. Em 1995, Andrade et al. realizaram um estudo em modelos animais com imuno-histoquímica e microscopia eletrônica, evidenciando que os adipócitos podem ser infectados pelo *T. cruzi* e até atuar como reservatórios para possíveis reativações da doença.^13^ Combs et al. (2005), ao realizarem um estudo em ratos infectados na fase aguda da DC, demonstraram que os adipócitos infectados com *T. cruzi* apresentam alterações na secreção de adipocitocinas. Observou-se uma redução nos níveis de adiponectina e leptina, além de um aumento nos níveis de interleucina-6 e TNF-ɑ.^14^ Em outro estudo, realizado por Nagajyothi et al. (2008), foi observado um aumento na expressão de citocinas pró-inflamatórias, incluindo interleucina-6 e TNF-ɑ, em adipócitos cultivados infectados com *T. cruzi* .^15^ Recentemente, Ferreira et al. demonstraram a persistência de *T. cruzi* em adipócitos de indivíduos com cardiopatia chagásica (CC) crônica.^16^ As alterações na função dos adipócitos causadas pela infecção por Chagas podem contribuir para a fisiopatologia da CC.^17 , 18^

Em pacientes com DC crônica, identificamos previamente níveis reduzidos de leptina e elevados de adiponectina na CC, em comparação com um grupo controle e outras formas de DC.^19 , 20^

Quanto ao metabolismo da glicose, há evidência de envolvimento pancreático em pacientes com DC aguda e crônica, com aumento no tamanho e número de ilhotas pancreáticas, infiltrado inflamatório, fibrose e despovoamento neuronal pancreático.^21 - 23^ Alguns estudos envolvendo pacientes crônicos demonstraram uma resposta reduzida de insulina à sobrecarga de glicose, tanto oral quanto venosa, sugerindo uma diminuição da secreção de insulina devido à denervação autonômica ou à lesão pancreática.^24 - 26^

Não há estudos que avaliem os níveis de adipocitocinas e insulina em indivíduos com DCA. Portanto, o objetivo deste estudo foi avaliar os níveis de adiponectina, leptina e insulina antes e após o tratamento da DCA e correlacioná-los com a avaliação do SNA e da função cardíaca por meio de ecocardiografia.

## Métodos

### Desenho do estudo

Trata-se de um estudo longitudinal com indivíduos avaliados na fase aguda da DC, antes e após um ano de tratamento etiológico com benznidazol.

### Área de estudo

O estudo foi realizado em Manaus (AM), em um centro terciário de atendimento especializado em doenças infecciosas. O recrutamento ocorreu de 28 de julho de 2017 a 28 de julho de 2018.

### Seleção de pacientes

Vinte e oito indivíduos foram divididos em dois grupos: grupo controle (GC) e grupo com doença de Chagas aguda (DCA). O grupo controle foi composto por 15 indivíduos saudáveis com sorologia não reativa para DC, enquanto o grupo com DCA incluiu 13 indivíduos com resultado positivo no exame parasitológico direto (teste de esfregaço sanguíneo espesso). Todos os pacientes no grupo com DCA foram tratados com 5-7 mg/kg de benznidazol (Rochagan^®^) durante um período de 60 dias.

Os critérios de exclusão incluíram infarto do miocárdio prévio, doença arterial coronariana, valvopatia cardíaca moderada ou grave (avaliada por exame clínico e ecocardiografia com Doppler), hipertensão arterial, diabetes mellitus, bloqueio atrioventricular avançado, marca-passo, doenças da tireoide, doença pulmonar obstrutiva crônica e IC nas classes funcionais III e IV pela classificação da New York Heart Association (NYHA). Todos os participantes assinaram um termo de consentimento esclarecido por escrito e o estudo foi aprovado pelo Comitê de Ética em Pesquisa da Universidade do Estado do Amazonas.

### Estudo eletrocardiográfico e ecocardiográfico

Todos os participantes foram submetidos a um ECG de 12 derivações (10 mm/mV e 25 mm/s) e a uma ecocardiografia transtorácica (ETT) completa, realizada com o aparelho de ultrassom GE Vivid 7, com software AFI ( *Automated Function Imaging* ) para análise de strain. Foram realizadas medições do ventrículo esquerdo (VE) guiadas por modo M bidimensional em visualização axial, e os volumes finais diastólico e sistólico do VE, átrios esquerdo e direito, foram obtidos a partir da visualização apical de 4 câmaras. A fração de ejeção do ventrículo esquerdo (FEVE) foi determinada pelo método biplano de Simpson modificado. O movimento regional da parede do VE e a ecocardiografia com strain foram avaliados com base no modelo de segmentação de 17 segmentos, e cada um foi confirmado em várias visualizações, com as seguintes categorias: normal ou hiperquinesia, hipocinesia, acinesia, discinesia e aneurismático. A função diastólica foi avaliada por Doppler de onda pulsada das velocidades de entrada transmitral, com o volume de amostra das pontas dos folhetos da valva mitral, e pela imagem Doppler tecidual do anel mitral septal e lateral, ambas na visualização apical de 4 câmaras. As regurgitações mitral, tricúspide, aórtica e pulmonar foram avaliadas qualitativamente.

### Avaliação da função do sistema nervoso autônomo

A função do SNA foi avaliada pela variabilidade da frequência cardíaca (VFC) nos domínios de tempo e frequência por meio do modelo de Transformada Rápida de Fourier. Os indivíduos foram avaliados por um período de cinco minutos em repouso, na posição de decúbito dorsal horizontal, com o uso de um monitor de frequência cardíaca (POLAR, modelo V800). Os seguintes índices foram calculados: Potência total, componente de baixa frequência (BF) em valores absolutos de potência (ms^2^) e em unidades normalizadas (u.n.), componente de alta frequência (AF) em valores absolutos de potência (ms^2^) e em unidades normalizadas (u.n.), e a razão BF/AF. O aumento da componente BF e da razão BF/AF foi interpretado como uma predominância da atividade simpática. O aumento da componente AF e a redução da razão BF/AF foram interpretados como uma predominância da atividade parassimpática.^20^

A fórmula a seguir foi utilizada para o cálculo das componentes BF e AF em unidades normalizadas (u.n.): - Componente BF ou AF em valores absolutos / (potência total – componente de frequência muito baixa) x 100.

Os pacientes foram orientados a não ingerir estimulantes, como café, chá, refrigerantes e bebidas alcoólicas, no dia anterior e no dia dos exames. Foi calculada a média das componentes espectrais obtidas nos tempos definidos. Foram excluídos ciclos cardíacos com variação superior a 25% em relação ao ciclo anterior, como forma de eliminar as alterações consequentes das extrassístoles ventriculares e supraventriculares. Foram avaliadas somente as leituras com pelo menos 85% de batimentos sinusais.

### Medições laboratoriais

O sangue venoso (10 mL) foi coletado por punção no membro superior após um período de 12 horas de jejum. O sangue foi centrifugado e o soro, armazenado a -70 °C para análises posteriores.

Os participantes foram submetidos a medições de níveis séricos de adiponectina, insulina e leptina por meio de um ensaio imunoenzimático (ELISA).

A insulina sérica foi medida utilizando um kit disponível comercialmente (Millipore, St. Charles, Missouri, EUA). A sensibilidade do kit era de 1 μU/mL, com o intervalo de referência entre 31 e 65 μU/mL. As medições foram realizadas em duplicata, apresentando um coeficiente de variação de 4,3%. A leptina sérica foi medida utilizando um kit disponível comercialmente (Millipore, St. Charles, Missouri, EUA). A sensibilidade do kit era de 0,78 ng/mL, com o intervalo de referência entre 12,9 e 26,8 ng/mL. As medições foram realizadas em duplicata, apresentando um coeficiente de variação de 7,3%. Os níveis séricos de adiponectina foram medidos utilizando um kit disponível comercialmente (Millipore, St. Charles, Missouri, EUA). A sensibilidade do kit era de 100 ng/mL, com o intervalo de referência entre 7.000 e 14.500 ng/mL. As medições foram realizadas em duplicata, apresentando um coeficiente de variação de 3,1%.

### Análise estatística

As medições da ETT e da VFC foram descritas de acordo com os períodos pré e pós-tratamento, utilizando médias e desvios padrão. As medições laboratoriais foram descritas utilizando medianas e intervalos interquartis devido à distribuição assimétrica dos dados, avaliada pelo teste de normalidade de Shapiro-Wilk. As variáveis categóricas foram descritas em percentuais. A comparação entre os períodos pré e pós-tratamento foi realizada por meio do teste t pareado ou do teste de Wilcoxon (não paramétrico), caso a normalidade das diferenças não fosse atendida.

Para as medições laboratoriais, também foram realizadas comparações entre o grupo pré-tratamento e o grupo controle, assim como entre o grupo pós-tratamento e o grupo controle. Os resultados também foram apresentados em gráficos do tipo Box Plot.

Para avaliar a correlação entre as variáveis de interesse, utilizamos gráficos de dispersão e apresentamos a correlação de Spearman.

Os testes de hipótese consideraram o nível de significância de 5%. As análises foram realizadas com o auxílio do software R (R Core Team, 2020).

### Considerações éticas

Este estudo foi aprovado pelo Comitê de Ética em Pesquisas com Seres Humanos da Fundação de Medicina Tropical Heitor Vieira Dourado-Amazonas em 28 de julho de 2017 sob o número 2.191.571, CAAE 69904017.9.0000.0005, e realizado de acordo com a Resolução N^o^ 466/12 do Conselho Nacional de Saúde do Brasil e as diretrizes éticas da Declaração de Helsinque de 1975. Todos os participantes assinaram o termo de consentimento esclarecido padronizado, aprovado anteriormente.

## Resultados

### Características clínicas

Treze pacientes com DCA, com idade média de 45,3 ± 23,7 anos, sendo 46,1% do sexo masculino, foram avaliados e comparados com 15 indivíduos do grupo controle, com idade média de 44,2 ± 7,3 anos, sendo 40% do sexo masculino.

Não foram observadas diferenças significativas nos grupos quanto a idade, sexo, peso e índice de massa corporal. As características físicas e hemodinâmicas basais dos pacientes com DC e dos indivíduos saudáveis do grupo controle são apresentadas na Tabela 1 .

**Tabela 1:** – Idade, peso e IMC conforme o grupo com doença de Chagas aguda (DCA) e o grupo controle (CG)

	DCA (n = 13)	GC (n = 15)	Valor de p*
Idade (anos)	45,3 ± 23,7	44,2 ± 7,3	0,872
Peso (kg)	63,7 ± 12,0	65,4 ± 6,9	0,659
IMC (kg/m^2^)	23,65 ± 2,5	23,9 ± 1,2	0,452

Os valores são expressos como média ± DP. IMC: índice de massa corporal. *Teste t pareado.

Todos os pacientes no grupo com DCA apresentaram sintomas de síndrome infecciosa, sendo a febre o sintoma mais comum (100%). Nenhum paciente teve sintomas cardíacos.

Antes do tratamento, 38,4% dos pacientes apresentaram eletrocardiograma normal, ao passo que, após o tratamento, 53,8% deles apresentaram eletrocardiograma normal. O distúrbio mais comum na fase pré-tratamento foi a repolarização ventricular, observada em 23% dos casos, ao passo que, na fase pós-tratamento, o bloqueio divisional anterossuperior foi o mais frequente, também em 23% dos casos.

### Medições ecocardiográficas e do SNA

O strain longitudinal global (SLG) pela ETT e os parâmetros de ETT, assim como a VFC dos pacientes antes e após o tratamento, foram comparados sem que fossem detectadas alterações significativas. Dos 13 pacientes recrutados, um não compareceu à consulta de acompanhamento. Esses parâmetros são indicados nas Tabelas 2 e 3 .

**Tabela 2:** – Medições ecocardiográficas conforme os períodos pré e pós-tratamento

	Pré-tratamento (n = 12)		Pós-tratamento (n = 12)		Valor de p*
	Média	DP	Média	DP	
DDFVE	48,10	5,12	48,40	6,02	0,756
VDFVE	109,00	29,90	112,00	30,70	0,613
FEVE	71,50	10,70	75,40	5,42	0,099
DDFVD	17,80	3,28	17,00	3,30	0,258
Relação E-e’	6,49	1,54	7,66	2,26	0,120
IPM-VD	0,40	0,22	0,35	0,06	0,414
IPM-VE	0,34	0,13	0,34	0,17	0,974
TAPSE*	19,80	4,15	20,00	4,18	0,848
SLG%	-19,9	4,2	-21,2	2,8	0,175

DDFVE: diâmetro diastólico final do ventrículo esquerdo; VDFVE: volume diastólico final do ventrículo esquerdo; FEVE: fração de ejeção do ventrículo esquerdo; DDFVD: diâmetro diastólico final do ventrículo direito; IPM-VD: índice de performance miocárdica do ventrículo direito; IPM-VE: índice de performance miocárdica do ventrículo esquerdo. *Teste t pareado.

**Tabela 3:** – Medições da variabilidade da frequência cardíaca conforme os períodos pré e pós-tratamento

VFC	Pré-tratamento (n = 12)		Pós-tratamento (n = 11)		Dif.	Valor de p*	IC de 95%	
	Média	DP	Média	DP			Inf.	Sup.
FC	80,70	7,45	70,60	12,40	10,10	0,048	0,09	20,10
RMSSD	22,30	19,60	27,60	13,60	-5,18	0,441	-19,60	9,21
SDNN	21,10	15,20	27,70	13,00	-6,37	0,231	-17,50	4,76
PT	542,00	655,00	849,00	841,00	-284,00	0,299	-86,00	293,00
BF ms^2^ *	240,00	243,00	408,00	359,00	-154,00	0,175	-389,00	81,20
AF ms^2^	256,00	348,00	366,00	434,00	-101,00	0,474	-403,00	202,00
AF un	42,90	17,20	45,20	14,50	-3,98	0,554	-18,50	10,50
BF AF	2,30	3,38	1,38	0,76	1,07	0,337	-1,30	3,44
BF un	56,70	17,50	54,60	14,50	3,85	0,566	-10,60	18,30

Os valores são expressos como média ± DP. FC: Frequência cardíaca; PT: potência total; BF: baixa frequência; AF: alta frequência; ms^2^: milissegundos quadrados; u.n.: unidades normalizadas. * Teste t pareado.

### Medições laboratoriais

Não houve diferenças significativas entre os grupos nos níveis de leptina e adiponectina. As principais diferenças encontradas foram nos níveis de insulina ( Figura Central ).

A Tabela 4 apresenta a comparação entre os períodos pré e pós-tratamento e o valor de p da comparação pareada entre os grupos. Observa-se que os valores de insulina no período pré-tratamento eram significativamente superiores aos do momento pós-tratamento.

**Tabela 4:** – Medições laboratoriais conforme os períodos pré e pós-tratamento

	Pré- tratamento (n=11)	Pós-tratamento (n=11)	Valor de p em Wilcox
Adiponectina (ng/mL)	6,9 [6,9; 6,9]	6,9 [6,8; 6,9]	0,646
Leptina (ng/mL)	5,4 [4,0; 5,7]	5,6 [5,5; 5,7]	0,293
Insulina (μU/mL)	3,1 [1,1; 4,1]	0,8 [0,7, 1,4]	0,034

Os valores são expressos como mediana [intervalo interquartil].

Não observamos diferenças significativas nas medições laboratoriais entre os grupos pré-tratamento de DC e o grupo controle ( Tabela 5 ). Entretanto, ao comparar os níveis de insulina entre o grupo pós-tratamento de DC e o grupo controle ( Tabela 6 ), observamos valores significativamente mais baixos no grupo pós-tratamento.

**Tabela 5:** – Medições laboratoriais conforme o grupo de pré-tratamento de DC e o grupo controle

	Pré-tratamento de DCA (n = 11)	Grupo controle (n = 15)	Valor de p
Adiponectina (ng/mL)	6,9 [6,9; 6,9]	6,8 [6,8; 6,8]	0,068
Leptina (ng/mL)	5,4 [4,0; 5,7]	5,6 [5,4; 5,6]	0,422
Insulina (μU/mL)	3,1 [1,1; 4,1]	4,1 [3,2; 4,9]	0,089

Os valores são expressos como mediana [intervalo interquartil].

**Tabela 6:** – Medições laboratoriais conforme o grupo de pós-tratamento de DC e o grupo controle

	Pós-tratamento de DCA (n = 11)	Grupo controle (n = 15)	Valor de p
Adiponectina (ng/mL)	6,9 [6,8; 6,9]	6,8 [6,8; 6,8]	0,054
Leptina (ng/mL)	5,6 [5,5; 5,7]	5,6 [5,4; 5,6]	0,388
Insulina (μU/mL)	0,8 [0,7; 1,4]	4,1 [3,2; 4,9]	< 0,001

Os valores são expressos como mediana [intervalo interquartil].

**Figura 1:**
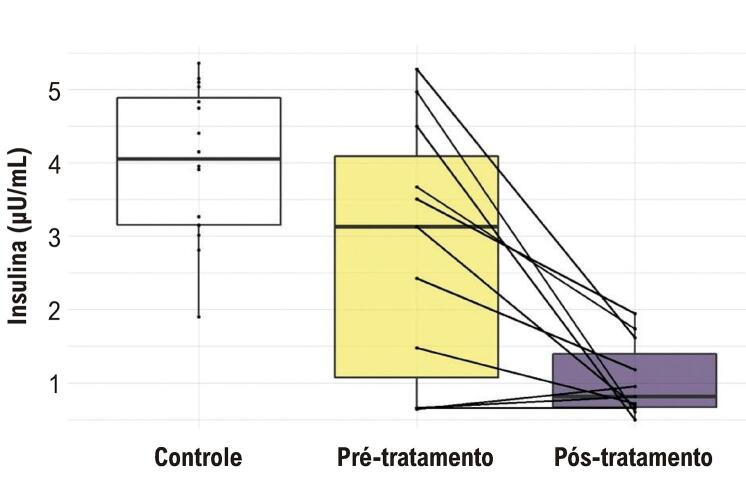
– Gráfico do tipo Box Plot dos níveis de insulina em cada grupo do estudo.

Na Figura 1 , são apresentados os gráficos do tipo Box Plot dos níveis de insulina em cada grupo do estudo. Os gráficos mostram a distribuição das variáveis em cada grupo. A linha no meio da caixa representa a mediana, a linha inferior indica o primeiro quartil e a linha superior indica o terceiro quartil. Nas figuras, também inserimos os pontos que correspondem aos valores de insulina em cada grupo e, entre os grupos pré e pós-tratamento, adicionamos uma linha para destacar o comportamento da variável entre ambos os períodos. Observamos valores mais elevados no grupo controle em comparação com os grupos com DC. Ao comparar os grupos com DC, observamos uma redução nos valores do período pré para o pós-tratamento.

### Correlações

Nenhuma correlação significativa foi detectada entre os níveis de adiponectina, leptina e insulina (pré e pós-tratamento) e as medições da função cardíaca (por meio do ecocardiograma) e da função autonômica ( Tabela 7 ).

**Tabela 7:** – Matriz de correlações entre as medições de ETT, Strain, Holter, VFC e MRI e as medições laboratoriais

Medições	Pré-tratamento			Pós-tratamento		
	Adiponectina	Leptina	Insulina	Adiponectina	Leptina	Insulina
DDFVE	0,10	0,10	0,27	-0,24	-0,36	0,14
VDFVE	0,13	0,05	0,29	-0,29	-0,40	0,12
FEVE	-0,46	0,46	-0,21	0,47	0,27	0,18
DDFVD	0,22	-0,27	-0,51	0,15	-0,10	0,15
Relação E-e’	-0,39	0,17	-0,05	0,62	0,18	0,58
IPM-VE	0,37	-0,18	0,45	-0,25	-0,32	-0,01
IPM-VD	0,08	0,20	-0,11	-0,44	-0,84	-0,29
TAPSE	0,26	-0,10	-0,30	0,34	0,07	0,08
SLG (%)	0,05	-0,20	0,47	-0,46	-0,03	-0,20
SDNN	-0,35	-0,19	-0,42	-0,29	0,23	-0,19
SDANN	-0,30	0,04	-0,48	-0,30	0,20	-0,29
SDNNI	-0,55	-0,25	-0,41	-0,40	0,08	-0,27
RMSSD	-0,79	-0,18	-0,38	-0,07	0,11	-0,26
PNN50	-0,79	-0,12	-0,39	0,01	0,27	-0,15
FC média	0,39	0,35	0,33	-0,24	0,00	-0,02
RMSSD	-0,15	-0,21	0,25	0,30	0,58	-0,19
SDNN	-0,24	-0,07	0,14	0,15	0,65	-0,15
PT	-0,25	-0,14	0,05	0,15	0,68	-0,15
BF ms^2^	-0,30	-0,19	0,02	0,04	0,53	-0,15
AF ms^2^	-0,18	-0,18	0,27	0,26	0,72	-0,22
BF un	-0,02	0,09	-0,37	-0,41	-0,20	-0,02
AF un	0,09	-0,05	0,36	0,41	0,20	0,02
BF AF	0,03	0,05	-0,34	-0,41	-0,20	0,04

DDFVE: diâmetro diastólico final do ventrículo esquerdo; VDFVE: volume diastólico final do ventrículo esquerdo; FEVE: fração de ejeção do ventrículo esquerdo; DDFVD: diâmetro diastólico final do ventrículo direito; IPM-VD: índice de performance miocárdica do ventrículo direito; IPM-VE: índice de performance miocárdica do ventrículo esquerdo; FC: frequência cardíaca; PT: potência total; BF: baixa frequência; AF: alta frequência; ms^2^: milissegundos quadrados; u.n.: unidades normalizadas.

## Discussão

Os níveis de insulina foram mais baixos no grupo com DCA em comparação com o grupo controle, tanto antes quanto após o tratamento, e apresentaram redução na fase pós-tratamento em comparação com a fase pré-tratamento.

Mediadores metabólicos, como adiponectina, leptina e insulina, foram analisados exclusivamente em humanos na fase crônica da DC. Na fase aguda, esses mediadores foram estudados apenas em modelos animais. Em publicações anteriores, nosso grupo demonstrou níveis reduzidos de leptina e insulina e níveis elevados de adiponectina em pacientes na fase crônica da DC.^19 , 20 , 27^

Nas últimas décadas, observou-se um aumento de casos agudos de DC, principalmente por transmissão oral, e com maior incidência na Amazônia brasileira. Esses casos apresentaram comprometimento cardíaco leve, com alterações no eletrocardiograma, no ecocardiograma e na ressonância magnética cardíaca.^3 , 28^

A principal alteração eletrocardiográfica encontrada foi a alteração da repolarização ventricular, observada em cerca de 30% dos casos, e a principal alteração ecocardiográfica foi o derrame pericárdico, observado em cerca de 10% dos casos. Esses pacientes, em sua maioria, apresentam melhora nas alterações após o tratamento e têm uma boa evolução clínica, sem progressão para IC.^3^

Entretanto, ao avaliar mais especificamente a função cardíaca e a morfologia com ressonância magnética cardíaca, são observadas alterações subclínicas, como a detecção de lesão miocárdica por meio do realce tardio do miocárdio em 58% dos casos.^28^

Pouco se sabe sobre a evolução a longo prazo dos pacientes tratados na fase aguda da DC ou em relação às alterações inflamatórias, metabólicas e do SNA nesses pacientes.

Este estudo foi o primeiro a avaliar os marcadores metabólicos, como adiponectina, leptina e insulina, nas fases pré e pós-tratamento da DCA em humanos. Não foram observadas diferenças significativas nos níveis de adipocitocina, adiponectina e leptina entre o grupo com DCA e o grupo controle, nem entre as fases pré e pós-tratamento. As principais alterações encontradas no presente estudo ocorreram nos níveis de insulina, a qual apresentou uma redução no grupo controle em comparação com os períodos pré e pós-tratamento.

A maioria dos estudos demonstra níveis reduzidos de insulina ou disfunção insulínica em pacientes chagásicos crônicos. Guariento et al. mostraram uma resposta insulínica reduzida à sobrecarga oral de glicose, sugerindo diminuição da secreção ou aumento da sensibilidade à insulina.^25^

Outro estudo demonstrou uma resposta insulínica reduzida à sobrecarga venosa de glicose, sugerindo um quadro hipoinsulinêmico em pacientes chagásicos devido à denervação ou à lesão pancreática.^24^ Em 2012, Silva et al. publicaram um estudo com teste de tolerância à insulina e observaram que pacientes chagásicos apresentaram uma resposta hemodinâmica inferior ao aumento da pressão arterial e da frequência cardíaca, quando comparados ao grupo controle. Essa resposta inferior sugere que a denervação simpática encontrada na DC compromete a resposta à ação da insulina.^29^ Nagajyothi et al., em estudo com modelo animal, observaram uma secreção reduzida de insulina na fase aguda da DC, com persistência até a fase crônica, provavelmente devido à inflamação do pâncreas e disfunção das células ß pancreáticas.^30^

Santos et al. (1999) relataram uma frequência mais alta de diabetes mellitus e hiperglicemia em mulheres com CC em comparação com os grupos controle, tanto na forma digestiva quanto na forma indeterminada. Eles sugeriram como explicação uma atividade simpática excessiva decorrente da denervação parassimpática e/ou hipoinsulinemia devido ao comprometimento funcional e anatômico do pâncreas.^31^

É importante destacar que pacientes com IC de outras etiologias apresentam níveis elevados de insulina e resistência à insulina.^32 , 33^ Portanto, a redução nos níveis de insulina encontrada em nosso estudo pode indicar a presença de um fator protetor contra a resistência à insulina ou fatores que interferem na secreção e/ou ação da insulina na DC.

Existem poucos estudos que avaliam os níveis de insulina em doenças infecciosas. Um desses estudos mostra aumento da resistência à insulina^34^ e outros demonstram comprometimento do pâncreas com perda de células beta pancreáticas e redução na expressão local de insulina.^35 , 36^ Sendo assim, os achados do nosso estudo podem ser atribuídos a alterações peculiares associadas à DC, como a disfunção autonômica, que interfere na secreção de insulina pelo pâncreas (denervação pancreática). Ademais, a fase aguda da DC pode causar alterações morfológicas e funcionais no pâncreas, resultando no comprometimento crônico,^23 , 27^ o que poderia explicar a presença de níveis reduzidos de insulina mesmo após o tratamento. Pesquisas futuras que investiguem marcadores específicos de lesão pancreática e índices de resistência à insulina podem proporcionar uma compreensão mais aprofundada desses mecanismos.

Este estudo foi pioneiro ao mostrar que a redução nos níveis de insulina começa na fase aguda pré-tratamento e se intensifica ainda mais na fase pós-tratamento da DCA. Os possíveis mecanismos para essa redução podem ser discutidos como mecanismos compensatórios relacionados ao desenvolvimento da CC, à presença de disfunção autonômica, à atividade inflamatória exacerbada e/ou ao comprometimento pancreático.

Neste grupo de pacientes, em função da ausência de correlações entre as variáveis de avaliação do SNA e a função cardíaca é fundamental estudar mais detalhadamente as correlações dos marcadores metabólicos com as citocinas inflamatórias e os marcadores de comprometimento pancreático. Além disso, são necessários mais estudos para avaliar a resistência à insulina a fim de detalhar melhor os mecanismos fisiopatológicos e as repercussões clínicas.

A limitação deste estudo foi o número reduzido de pacientes, principalmente devido à dificuldade de acesso à região Amazônica. Em razão disso, não foi realizado o cálculo do tamanho da amostra, optando-se por uma amostra de conveniência. No futuro, a análise de um maior número de pacientes poderá proporcionar uma compreensão mais aprofundada dos mecanismos e das repercussões clínicas das alterações observadas neste estudo.

## Conclusão

Os níveis de insulina foram mais baixos no grupo com DCA em comparação com o grupo controle, tanto antes quanto após o tratamento, e apresentaram redução na fase pós-tratamento em comparação com a fase pré-tratamento. Não houve diferença nos níveis de adipocitocinas, leptina e adiponectina entre os grupos. Os níveis de adipocitocinas e insulina não apresentaram correlação significativa com os parâmetros de função cardíaca e do SNA.

## References

[1] World Health Organization (2012). Research priorities for Chagas Disease, Human African Trypanosomiasis and Leishmaniasis. World Health Organ Tech Rep Ser.

[2] Gascon J, Bern C, Pinazo MJ (2010). Chagas Disease in Spain, the United States and Other Non-Endemic Countries. Acta Trop.

[3] Ortiz JV, Pereira BVM, Couceiro KDN, Silva MRHS, Doria SS, Silva PRLD (2019). Cardiac Evaluation in the Acute Phase of Chagas' Disease with Post-Treatment Evolution in Patients Attended in the State of Amazonas, Brazil. Arq Bras Cardiol.

[4] Marin-Neto JA, Cunha-Neto E, Maciel BC, Simões MV (2007). Pathogenesis of Chronic Chagas Heart Disease. Circulation.

[5] Wajchenberg BL, Nery M, Cunha MR, Silva ME (2009). Adipose Tissue at the Crossroads in the Development of the Metabolic Syndrome, Inflammation and Atherosclerosis. Arq Bras Endocrinol Metabol.

[6] Antoniades C, Antonopoulos AS, Tousoulis D, Stefanadis C (2009). Adiponectin: From Obesity to Cardiovascular Disease. Obes Rev.

[7] Guzik TJ, Mangalat D, Korbut R (2006). Adipocytokines - Novel Link between Inflammation and Vascular Function?. J Physiol Pharmacol.

[8] Berg AH, Scherer PE (2005). Adipose Tissue, Inflammation, and Cardiovascular Disease. Circ Res.

[9] George J, Patal S, Wexler D, Sharabi Y, Peleg E, Kamari Y (2006). Circulating Adiponectin Concentrations in Patients with Congestive Heart Failure. Heart.

[10] Dieplinger B, Gegenhuber A, Poelz W, Haltmayer M, Mueller T (2009). Prognostic Value of Increased Adiponectin Plasma Concentrations in Patients with Acute Destabilized Heart Failure. Clin Biochem.

[11] Schulze PC, Kratzsch J, Linke A, Schoene N, Adams V, Gielen S (2003). Elevated Serum Levels of Leptin and Soluble Leptin Receptor in Patients with Advanced Chronic Heart Failure. Eur J Heart Fail.

[12] Filippatos GS, Tsilias K, Venetsanou K, Karambinos E, Manolatos D, Kranidis A (2000). Leptin Serum Levels in Cachectic Heart Failure Patients. Relationship with Tumor Necrosis Factor-Alpha System. Int J Cardiol.

[13] Andrade ZA, Silva HR (1995). Parasitism of Adipocytes by Trypanosoma Cruzi. Mem Inst Oswaldo Cruz.

[14] Combs TP, Nagajyothi, Mukherjee S, Almeida CJ, Jelicks LA, Schubert W (2005). The Adipocyte As an Important Target Cell for Trypanosoma Cruzi Infection. J Biol Chem.

[15] Nagajyothi F, Desruisseaux MS, Thiruvur N, Weiss LM, Braunstein VL, Albanese C (2008). Trypanosoma Cruzi Infection of Cultured Adipocytes Results in an Inflammatory Phenotype. Obesity.

[16] Ferreira AV, Segatto M, Menezes Z, Macedo AM, Gelape C, Andrade LO (2011). Evidence for Trypanosoma Cruzi in Adipose Tissue in Human Chronic Chagas Disease. Microbes Infect.

[17] Nagajyothi F, Desruisseaux MS, Weiss LM, Chua S, Albanese C, Machado FS (2009). Chagas Disease, Adipose Tissue and the Metabolic Syndrome. Mem Inst Oswaldo Cruz.

[18] Tanowitz HB, Jelicks LA, Machado FS, Esper L, Qi X, Desruisseaux MS, Chua SC (2011). Adv Parasitol.

[19] Fernandes F, Dantas S, Ianni BM, Ramires FJ, Buck P, Salemi VM (2007). Leptin Levels in Different Forms of Chagas' Disease. Braz J Med Biol Res.

[20] Barbosa-Ferreira JM, Mady C, Ianni BM, Lopes HF, Ramires FJ, Salemi VM (2015). Dysregulation of Autonomic Nervous System in Chagas' Heart Disease Is Associated with Altered Adipocytokines Levels. PLoS One.

[21] Rocha A, Oliveira LC, Alves RS, Lopes ER (1998). Pancreatic Neuronal Loss in Chronic Chagas' Disease Patients. Rev Soc Bras Med Trop.

[22] Saldanha JC, Santos VM, Reis MA, Cunha DF, Teixeira VPA (2001). Morphologic and Morphometric Evaluation of Pancreatic Islets in Chronic Chagas' Disease. Rev Hosp Clin Fac Med Sao Paulo.

[23] Santos VM, Lima MA, Cabrine-Santos M, Marquez DS, Pereira GA, Lages-Silva E (2004). Functional and Histopathological Study of the Pancreas in Hamsters (Mesocricetus Auratus) Infected and Reinfected with Trypanosoma Cruzi. Parasitol Res.

[24] Oliveira LC, Juliano Y, Novo NF, Neves MM (1993). Blood Glucose and Insulin Response to Intravenous Glucose by Patients with Chronic Chagas' Disease and Alcoholism. Braz J Med Biol Res.

[25] Guariento ME, Saad MJ, Muscelli EO, Gontijo JA (1993). Heterogenous Insulin Response to an Oral Glucose Load by Patients with the Indeterminate Clinical Form of Chagas' Disease. Braz J Med Biol Res.

[26] Guariento ME, Olga E, Muscelli A, Gontijo JA (1994). Chronotropic and Blood Pressure Response to Oral Glucose Load in Chagas' Disease. Sao Paulo Med J.

[27] Dabarian AL, Mady C, Barbosa-Ferreira JM, Ianni BM, Hotta VT, Ramires FJA (2019). Dysregulation of Insulin Levels in Chagas Heart Disease is Associated with Altered Adipocytokine Levels. Can J Physiol Pharmacol.

[28] Couceiro KDN, Ortiz JV, Silva MRHS, Sousa DRT, Andrade RC, Brandão ARJ (2022). Myocardial Injury in Patients with Acute and Subacute Chagas Disease in the Brazilian Amazon Using Cardiovascular Magnetic Resonance. J Am Heart Assoc.

[29] Silva CC, Santos CA, Mostarda C, Krieger EM, Lopes HF (2012). Blood Pressure, Metabolic and Autonomic Responses to Insulin and Intralipid® Infusion in Chagasic Patients. Arq Bras Cardiol.

[30] Nagajyothi F, Kuliawat R, Kusminski CM, Machado FS, Desruisseaux MS, Zhao D (2013). Alterations in Glucose Homeostasis in a Murine Model of Chagas Disease. Am J Pathol.

[31] Santos VM, Cunha SF, Teixeira VP, Monteiro JP, Santos JA, Santos TA (1999). Frequency of Diabetes Mellitus and Hyperglycemia in Chagasic and Non-Chagasic Women. Rev Soc Bras Med Trop.

[32] Schulze PC, Biolo A, Gopal D, Shahzad K, Balog J, Fish M (2011). Dynamics in Insulin Resistance and Plasma Levels of Adipokines in Patients with Acute Decompensated and Chronic Stable Heart Failure. J Card Fail.

[33] Leyva F, Anker SD, Egerer K, Stevenson JC, Kox WJ, Coats AJ (1998). Hyperleptinaemia in Chronic Heart Failure. Relationships with Insulin. Eur Heart J.

[34] Park SK, Cho YK, Park JH, Kim HJ, Park DI, Sohn CI (2010). Change of Insulin Sensitivity in Hepatitis C Patients with Normal Insulin Sensitivity: A 5-Year Prospective Follow-Up Study Variation of Insulin Sensitivity in HCV Patients. Intern Med J.

[35] Wu CT, Lidsky PV, Xiao Y, Lee IT, Cheng R, Nakayama T (2021). SARS-CoV-2 Infects Human Pancreatic ß Cells and Elicits ß Cell Impairment. Cell Metab.

[36] Deng W, Bao L, Song Z, Zhang L, Yu P, Xu Y (2024). Infection with SARS-CoV-2 Can Cause Pancreatic Impairment. Signal Transduct Target Ther.

