# Supplementary material for: Redução dos Níveis de Insulina em Pacientes com Doença de Chagas Aguda na Amazônia Brasileira Tratados com Benznidazol
Source: Arq Bras Cardiol. 2025 May 8;122(5):e20240621. [Article in Portuguese] doi: 10.36660/abc.20240621 (PMC12129477; doi:10.36660/abc.20240621)
Supplement: Translation

# Translation: Decreased Insulin Levels in Patients with Acute Chagas Disease from Brazilian Amazon treated with Benznidazole

**DOI:** 10.36660/abc.20240621i

**Authors:** João Marcos Barbosa-Ferreira, Jessica Vanina Ortiz, Fernanda Gallinaro Pessoa, Maria das Graças Vale Barbosa, Jorge Augusto de Oliveira Guerra, Kátia do Nascimento Couceiro, Mônica Regina Hosannah Silva e Silva, Matheus Martins Monteiro, Keila Cardoso Barbosa Fonseca, Felix Alvarez Ramires, Barbara Maria Ianni, Charles Mady, Fábio Fernandes

**Affiliations:** 1 Brazil Universidade do Estado do Amazonas, Manaus, AM – Brazil; 2 Brazil Programa de Pós-Graduação em Medicina Tropical – Universidade do Estado do Amazonas, Manaus, AM – Brazil; 3 Brazil Instituto do Coração do Hospital das Clínicas da Faculdade de Medicina da Universidade de São Paulo, São Paulo, SP – Brazil

**Keywords:** Insulin, Adipokines, Chagas Disease, Amazonian Ecosystem, Chagas Cardiomyopathy

## Abstract

**Background:**

In the Amazon region, there has been a significant increase in the number of acute Chagas disease (ACD) leading to electrocardiographic and echocardiographic abnormalities. The main pathophysiological mechanisms involved in Chagas cardiomyopathy (CCM) are microvascular dysfunction, Autonomic Nervous System (ANS) dysfunction, direct parasite aggression, and inflammatory activity. Chagas disease (CD) is a perfect example of inflammatory cardiomyopathy that might influence changes in metabolic parameters and ANS. Studies on the measurement of adipocytokines and insulin levels in humans with ACD are scarce.

**Objective:**

To evaluate adiponectin, leptin and insulin levels before and after the treatment of ACD with benznidazole and to correlate with ANS and cardiac function.

**Methods:**

Twenty-eight subjects were divided into groups: control group (CG), with 15 subjects, and ACD group, with 13 subjects. All subjects underwent a standard 12-lead ECG, comprehensive transthoracic echocardiographic, evaluation of autonomic function and serum adiponectin, insulin and leptin levels. A level of significance of 5% (p-value < 0,05) was used.

**Results:**

Insulin levels were lower in the ACD group both before and after the treatment when compared to the control group and lower in the post-treatment phase when compared to the pre-treatment phase. Adipocytokine, leptin and adiponectin levels showed no differences between groups.

**Conclusion:**

Insulin levels were lower in the ACD group both before and after the treatment when compared to the control group and lower in the post-treatment phase when compared to the pre-treatment phase. There was no significant correlation between adipocytokine and insulin levels with cardiac function and the ANS parameters.

## Introduction

Chagas disease (CD) occurs from the southern United States to Patagonia and affects around 8 million people in Latin America.^1^Moreover, CD is becoming increasingly important in non-endemic countries, such as the United States, Canada, some European countries, Japan, and Australia, due to the intensification of the migratory flow. In the United States, it is estimated that 300 thousand legal immigrants may be infected with the disease. Spain has the second largest prevalence of the disease, with around 40 to 60 thousand infected immigrants.^2^In Brazil, and more specifically in the Amazon region, , there has been a significant increase in the number of cases in recent decades, particularly in the acute form of the disease, transmitted by the consumption of contaminated regional fruit juices, including cases with cardiac impairment and electrocardiographic and echocardiographic alterations.^3^

Progression to myocardial dysfunction is the major cause of morbidity and mortality in CD and occurs in approximately 30% of patients. The main pathophysiological mechanisms involved in Chagas cardiomyopathy (CCM) are microvascular dysfunction, impairment of the Autonomic Nervous System (ANS), direct parasite aggression, and severe inflammatory activity.^4^Some of these mechanisms have the potential to also influence changes in metabolic parameters in patients with CD, such as autonomic dysfunction and inflammatory activity. Some of the most important mediators of metabolic activity are secreted from adipose tissue (adipocytokines) or are involved in glucose metabolism.

The adipocytokines are bioactive mediators produced by adipose tissue. The main adipocytokines are adiponectin and leptin. Adiponectin has beneficial anti-inflammatory and anti-atherogenic effects, as well as insulin-sensitizing action.^5 - 9^However, the role of adiponectin in cardiovascular disease is still controversial. Previous studies have shown that adiponectin levels are elevated in patients with systolic heart failure (HF), even predicting morbidity and mortality.^10 - 11^Leptin promotes pro-inflammatory and pro-thrombotic activities, neointimal proliferation, endothelial dysfunction, and induction of insulin resistance.^12^

*Trypanosoma cruzi* has a tropism for adipose tissue. In 1995, Andrade et al. carried out a study in animal models with immunohistochemistry and electron microscopy, demonstrating that adipocytes can be infected by *T. cruzi* and even serve as a reservoir for possible reactivations of the disease.^13^Combs et al. (2005), by performing a study involving rats infected in the acute phase of CD, demonstrated that adipocytes infected with *T. cruzi* exhibit changes in the secretion of adipocytokines. There was a decrease in the levels of adiponectin and leptin and an increase in the levels of interleukin-6 and TNF-ɑ.^14^Another study, carried out by Nagajyothi et al. (2008), demonstrated an increase in the expression of pro-inflammatory cytokines, including interleukin-6 and TNF-ɑ, in cultured adipocytes infected with *T. cruzi.* ^15^Recently, Ferreira et al. demonstrated the persistence of *T. cruzi* in adipocytes from humans with chronic Chagas heart disease (CHD).^16^Alterations in adipocyte function caused by Chagas infection may contribute to the pathophysiology of CHD.^17 , 18^

In patients with chronic CD, we have previously found reduced leptin levels and increased adiponectin levels in CCM when compared with a control group and other forms of CD.^19 , 20^

Regarding glucose metabolism, there is evidence of pancreatic involvement in patients with acute and chronic CD, with an increase in the size and number of pancreatic islets, inflammatory infiltrate, fibrosis, and pancreatic neuronal depopulation.^21 - 23^Some studies involving chronic patients have demonstrated a decreased insulin response to glucose overload, both oral and venous, suggesting reduced insulin secretion caused by autonomic denervation or pancreatic injury.^24 - 26^

There are no studies assessing adipocytokine and insulin levels in humans with ACD. Therefore, this study aimed to evaluate adiponectin, leptin and insulin levels before and after the treatment of ACD and to correlate these levels with the assessment of the ANS and cardiac function through echocardiography.

## Methods

### Study design

This is a longitudinal study involving subjects evaluated during the acute phase of CD, before treatment and one year after the etiologic treatment with benznidazole.

### Study Area

The study was carried out in the city of Manaus **,** state of Amazonas, Brazil, a tertiary care center specialized in infectious diseases. The recruitment period was from July 28^th^, 2017 to July 28^th^, 2018.

### Patient selection

Twenty-eight subjects were divided into two groups: a control group (CG) and an ACD group. The control group consisted of 15 healthy subjects with non-reactive serology for CD, and the ACD group included 13 subjects with a positive direct parasitological examination (thick blood smear test). All patients in the ACD group were treated with 5-7 mg/kg of benznidazole (Rochagan®) for 60 days.

Exclusion criteria were previous myocardial infarction, coronary artery disease, moderate or severe valvular heart disease (as assessed by clinical examination and Doppler echocardiography), arterial hypertension, diabetes mellitus, advanced atrioventricular block, pacemaker, thyroid diseases, chronic obstructive pulmonary disease, and HF in functional classes III and IV of the New York Heart Association (NYHA) classification. All subjects signed a written informed consent, and the study was approved by the Ethics Committee of the Amazonas State University.

### Electrocardiographic and echocardiographic study

All subjects underwent a standard 12-lead ECG (10 mm/mV and 25 mm/s), and a comprehensive transthoracic echocardiographic (TTE) was performed with Vivid 7 GE ultrasound machine with Automated Function Imaging software for strain analysis. Two-dimensional guided M-mode measurements of the left ventricle (LV) in short axis view and LV, left atrium and right atrium end-diastolic and end-systolic volumes were taken from the apical 4-chamber view. LV ejection fraction (LVEF) was calculated using a modified Simpson biplane method. LV regional wall motion and strain echo were evaluated based on the 17-segment model segmentation, and each segment was confirmed in multiple views as follows: normal or hyperkinesis, hypokinesis, akinesis, dyskinesis, and aneurysmal. Diastolic function was assessed from pulsed-wave Doppler of the transmitral inflow velocities, with the sample volume at the mitral valve leaflet tips, and from tissue Doppler imaging of the septal and lateral mitral annulus, both at the apical 4-chamber view. Mitral, tricuspid, aortic, and pulmonary regurgitations were qualitatively evaluated.

### Evaluation of autonomic nervous system function

ANS function was assessed by heart rate variability (HRV) in the time and frequency domain using the Fast Fourier Transform model. Subjects were assessed for five minutes at rest in the horizontal dorsal decubitus using a heart rate monitor (POLAR, model V800). The following indices were calculated: Total power, low-frequency (LFr) component in absolute values of power (ms^2^) and in normalized units (n.u.), high-frequency (HFr) component in absolute values of power (ms^2^) and in normalized units (n.u.), and the LFr/HFr ratio. The increase of the LF component and the LFr/HFr ratio were interpreted as a predominance of sympathetic activity. The increase in the HFr component and the reduction in the LFr/HFr ratio were interpreted as a predominance of parasympathetic activity.^20^

The following formula was used for the calculation of the LFr and HFr components in normalized units (n.u.): LFr or HFr component in absolute values / (total power–very low-frequency component) x 100.

Patients were instructed to abstain from taking any stimulants, such as coffee, tea, soft drinks, and alcoholic beverages, on the day before and on the day of the exams. The mean of the spectral components obtained at the established times was calculated. Cardiac cycles with a deviation of more than 25% from a previous one were excluded in order to eliminate the resulting alterations to ventricular and supraventricular extrasystoles. Only readings with at least 85% sinus beats were assessed.

### Laboratory measurements

Venous blood (10 ml) was collected by puncture in the upper limb after a 12-hour fast. The blood was centrifuged, and the serum was stored at -70°C for further analysis.

The subjects underwent serum adiponectin, insulin and leptin measurements by means of an enzyme-linked immunosorbent assay (ELISA).

Serum insulin levels were measured using a commercially available kit (Millipore, St. Charles, Missouri, USA). The sensitivity of the kit was 1 μU/mL, and the reference interval was from 31 to 65 μU/ml. Measurements were performed in duplicate with a coefficient of variation of 4.3%. Serum leptin levels were measured using a commercially available kit (Millipore, St. Charles, Missouri, USA). The sensitivity of the kit was 0.78 ng/mL, and the reference interval was from 12.9 to 26.8 ng/mL. Measurements were performed in duplicate with a coefficient of variation of 7.3%. Serum adiponectin levels were measured using a commercially available kit (Millipore, St. Charles, Missouri, USA). The sensitivity of the kit was 100 ng/mL, and the reference interval was from 7000 to 14,500 ng/mL. Measurements were performed in duplicate with a coefficient of variation of 3.1%.

### Statistical analysis

The TTE and HRV measurements were described by means and standard deviations, according to the pre- and post-treatment periods. Laboratory measurements were described using medians and interquartile ranges due to the asymmetric distribution of the data, as assessed by the Shapiro-Wilk normality test. Categorical variables were described using percentages. The comparison of pre- and post-treatment periods was performed using the paired T-test or Wilcoxon (non-parametric) test when the assumption of normality of the differences was not supported.

For the laboratory measurements, we also made comparisons of the pre-treatment groups with the control group and the post-treatment group with the control group. The results were also shown in Box Plot graphs.

To assess the correlation between the variables of interest, we used scatter plots and presented Spearman’s correlation.

Hypothesis tests were performed at the 5% significance level. Analyses were performed using R software (R Core Team, 2020).

### Ethical considerations

This study was approved by the Human Research Ethics Committee of Fundação de Medicina Tropical Heitor Vieira Dourado-Amazonas on July 28^th^, 2017, under No. 2.191.571, CAAE No. 69904017.9.0000.0005, and carried out in accordance with Resolution No. 466/12 of the Brazilian National Health Council and the ethical guidelines of the 1975 Declaration of Helsinki. All participants signed a standardized and previously approved informed consent.

## Results

### Clinical characteristics

Thirteen ACD patients with a mean age of 45.3 ± 23.7 years, of whom 46.1% were male, were evaluated and compared with 15 subjects in the control group with a mean age of 44.2 ± 7.3 years, of whom 40% were male.

There were no significant differences in age, sex, weight, and body mass index between the groups. Baseline physical and hemodynamic characteristics of patients with CD and healthy subjects in the control group are shown in Table 1 .

**Table 1:** – Age, weight, and BMI according to acute chagas disease (ACD) and control group (CG)

	ACD (n = 13)	CG (n = 15)	p-value*
Age (years)	45.3 ± 23.7	44.2 ± 7.3	0.872
Weight (kg)	63.7 ± 12.0	65.4 ± 6.9	0.659
BMI (kg/m^2^)	23.65 ± 2.5	23.9 ± 1.2	0.452

Values expressed as mean ± SD. BMI: body mass index.* Paired t-test.

All patients in the ACD group had symptoms of infectious syndrome, with fever being the most frequent symptom (100%). No patient had cardiac symptoms.

Electrocardiogram was normal for 38.4% of patients before treatment and for 53.8% of patients after treatment. The most frequent abnormality in the pre-treatment phase was ventricular repolarization, present in 23% of cases, and in the post-treatment phase was the anterosuperior divisional block, present in 23% of cases.

### Echocardiographic and ANS measurements

TTE global longitudinal strain (GLS) and TTE parameters, as well as the HRV of the patients before and after treatment, were compared without detecting significant alterations. Of the 13 patients recruited, one did not attend the follow-up visit. These parameters are described in Tables 2 and 3 .

**Table 2:** – Echocardiogram measurements according to pre- and post-treatment periods

	Pre-treatment (n = 12)		Post-treatment (n = 12)		p-value*
	Mean	SD	Mean	SD	
LVED	48.10	5.12	48.40	6.02	0.756
LVEDV	109.00	29.90	112.00	30.70	0.613
LVEF	71.50	10.70	75.40	5.42	0.099
RVED	17.80	3.28	17.00	3.30	0.258
E/E’ ratio	6.49	1.54	7.66	2.26	0.120
RVMPI	0.40	0.22	0.35	0.06	0.414
LVMPI	0.34	0.13	0.34	0.17	0.974
TAPSE*	19.80	4.15	20.00	4.18	0.848
GLS%	-19.9	4.2	-21.2	2.8	0.175

LVED: left ventricular end-diastolic diameter, LVEDV: left ventricular end-diastolic volume, LVEF: left ventricular ejection Fraction, RVED: right ventricular end-diastolic diameter; RVMPI: right ventricular myocardial performance index, LVMPI: left ventricular myocardial performance index. *Paired t-test.

**Table 3:** – Heart rate variability measurements according to pre- and post-treatment periods

HRV	Pre-treatment (n=12)		Post-treatment (n=11)		Dif.	p-value*	95% CI	
	Mean	SD	Mean	SD			Inf.	Sup.
HR	80.70	7.45	70.60	12.40	10.10	0.048	0.09	20.10
RMSSD	22.30	19.60	27.60	13.60	-5.18	0.441	-19.60	9.21
SDNN	21.10	15.20	27.70	13.00	-6.37	0.231	-17.50	4.76
TP	542.00	655.00	849.00	841.00	-284.00	0.299	-86.00	293.00
LF ms^2^*	240.00	243.00	408.00	359.00	-154.00	0.175	-389.00	81.20
HF ms^2^	256.00	348.00	366.00	434.00	-101.00	0.474	-403.00	202.00
HF un	42.90	17.20	45.20	14.50	-3.98	0.554	-18.50	10.50
LF HF	2.30	3.38	1.38	0.76	1.07	0.337	-1.30	3.44
LF n.u.	56.70	17.50	54.60	14.50	3.85	0.566	-10.60	18.30

Values expressed as mean ± SD. HR: heart rate; TP: total power; LF: low frequency; HF: high frequency; ms^2^: milliseconds squared; n.u.: normalized units. *Paired t-test.

### Laboratory measurements

There were no significant differences in leptin and adiponectin levels between groups. The main differences found were in insulin levels ( Central Illustration ).

Table 4 shows the comparison between the pre- and post-treatment periods and the p-value of the paired comparison between the groups. It is noted that the insulin values at the pre-treatment period were significantly higher than the post-treatment period.

**Table 4:** – Laboratory measurements according to pre- and post-treatment periods

	Pre- treatment (n=11)	Post-treatment (n=11)	Wilcox p-value
Adiponectin (ng/mL)	6.9 [6.9; 6.9]	6.9 [6.8; 6.9]	0.646
Leptin (ng/mL)	5.4 [4.0; 5.7]	5.6 [5.5; 5.7]	0.293
Insulin (μU/mL)	3.1 [1.1; 4.1]	0.8 [0.7. 1.4]	0.034

Values expressed as medians [interquartile range].

We did not observe significant differences in laboratory measurements between the pre-treatment and control groups ( Table 5 ). However, when comparing insulin levels in the post-treatment group and the control group ( Table 6 ), we observed significantly lower values in the post-treatment group.

**Table 5:** – Laboratory measurements according to Pre-Treatment groups and the Control group

	Pre-treatment of ACD (n=11)	Control Group (n=15)	p-value
Adiponectin (ng/mL)	6.9 [6.9; 6.9]	6.8 [6.8; 6.8]	0.068
Leptin (ng/mL)	5.4 [4.0; 5.7]	5.6 [5.4; 5.6]	0.422
Insulin (μU/mL)	3.1 [1.1; 4.1]	4.1 [3.2; 4.9]	0.089

Values expressed as medians [interquartile range]. ACD: acute Chagas disease.

**Table 6:** – Laboratory measurements according to Post-Treatment groups and the Control group

	Post-treatment of ACD (n=11)	Control Group (n=15)	p-value
Adiponectin (ng/mL)	6.9 [6.8; 6.9]	6.8 [6.8; 6.8]	0.054
Leptin (ng/mL)	5.6 [5.5; 5.7]	5.6 [5.4; 5.6]	0.388
Insulin (μU/mL)	0.8 [0.7; 1.4]	4.1 [3.2; 4.9]	< 0.001

Values expressed as medians [interquartile range]. ACD: acute Chagas disease.

Figure 1 shows the insulin box plots in each study group. Box plots show the distribution of variables in each group. The dash in the middle of the box shows the median, the bottom dash shows the first quartile, and the top dash shows the third quartile. In the figures, we also inserted the points corresponding to the insulin values in each group and, between the pre- and post-treatment groups, we added a line to highlight the behavior of the variable between the periods. We noticed higher values in the control group compared to the CD group. When comparing the CD groups, we noticed a decrease in the values from the pre- to the post-treatment moment.

**Figure 1:** – Box plot for insulin values in each study group.

### Correlations

No significant correlations were found between adiponectin, leptin and insulin (before and after treatment) levels and measures of cardiac function (via echocardiogram) and autonomic function ( Table 7 ).

**Table 7:** – Matrix of correlations between TTE, Strain, Holter, HRV, and MRI measurements with laboratory measurements

Measures	Pre-treatment			Post-treatment		
	Adiponectin	Leptin	Insulin	Adiponectin	Leptin	Insulin
LVED	0.10	0.10	0.27	-0.24	-0.36	0.14
LVEDV	0.13	0.05	0.29	-0.29	-0.40	0.12
LVEF	-0.46	0.46	-0.21	0.47	0.27	0.18
RVED	0.22	-0.27	-0.51	0.15	-0.10	0.15
E/E’ ratio	-0.39	0.17	-0.05	0.62	0.18	0.58
LVMPI	0.37	-0.18	0.45	-0.25	-0.32	-0.01
RVMPI	0.08	0.20	-0.11	-0.44	-0.84	-0.29
TAPSE	0.26	-0.10	-0.30	0.34	0.07	0.08
GLS %	0.05	-0.20	0.47	-0.46	-0.03	-0.20
SDNN	-0.35	-0.19	-0.42	-0.29	0.23	-0.19
SDANN	-0.30	0.04	-0.48	-0.30	0.20	-0.29
SDNNI	-0.55	-0.25	-0.41	-0.40	0.08	-0.27
RMSSD	-0.79	-0.18	-0.38	-0.07	0.11	-0.26
PNN50	-0.79	-0.12	-0.39	0.01	0.27	-0.15
HR mean	0.39	0.35	0.33	-0.24	0.00	-0.02
RMSSD	-0.15	-0.21	0.25	0.30	0.58	-0.19
SDNN	-0.24	-0.07	0.14	0.15	0.65	-0.15
TP	-0.25	-0.14	0.05	0.15	0.68	-0.15
LF ms^2^	-0.30	-0.19	0.02	0.04	0.53	-0.15
HF ms^2^	-0.18	-0.18	0.27	0.26	0.72	-0.22
LF n.u.	-0.02	0.09	-0.37	-0.41	-0.20	-0.02
HF n.u.	0.09	-0.05	0.36	0.41	0.20	0.02
LF HF	0.03	0.05	-0.34	-0.41	-0.20	0.04

LVED: left ventricular end-diastolic diameter; LVEDV: left ventricular end-diastolic volume; LVEF: left ventricular ejection fraction; RVED: right ventricular end-diastolic diameter; RVMPI: right ventricular myocardial performance index; LVMPI: left ventricular myocardial performance index; HR: heart rate; TP: total power; LF: low frequency; HF: high frequency; ms^2^: milliseconds squared; n.u.: normalized units.

## Discussion

Insulin levels were lower in the ACD group both before and after treatment when compared to the control group and lower in the post-treatment phase when compared to the pre-treatment phase.

Metabolic mediators such as adiponectin, leptin and insulin have been studied in humans only in the chronic phase of CD. In the acute phase of the disease, these mediators have only been studied in animal models. In previous publications, our group has shown decreased levels of leptin and insulin and increased levels of adiponectin in patients in the chronic phase of CD.^19 , 20 , 27^

In recent decades, there has been a resurgence of acute cases of CD, mainly by oral transmission and mainly in the Brazilian Amazon. These cases have shown mild cardiac involvement with electrocardiographic, echocardiographic and cardiac magnetic resonance alterations.^3 , 28^

The main electrocardiographic alteration found was the alteration of ventricular repolarization, present in about 30% of the cases, and the main echocardiographic alteration found was the pericardial effusion, present in about 10% of the cases. These patients, for the most part, show improvement in the alterations after treatment and have a good clinical evolution without progression to HF in most cases.^3^

However, when cardiac function and morphology are evaluated more specifically with cardiac magnetic resonance, subclinical changes are observed, such as the detection of myocardial injury through the detection of late myocardial enhancement in 58% of cases.^28^

Little is known about the long-term evolution of patients treated in the acute phase of CD or the inflammatory, metabolic and ANS changes in these patients.

This work was the first to evaluate metabolic markers, such as adiponectin, leptin and insulin, in the pre- and post-treatment phase of ACD in humans. There were no significant differences in adipocytokine, adiponectin and leptin levels between the group of patients with ACD and the control group, or between the pre- and post-treatment phases. The main alterations found in this study were in insulin levels, which showed a decrease in the control group compared to the pre-treatment and post-treatment phases.

Most studies demonstrate decreased insulin levels or impaired insulin function in patients with chronic CD. Guariento et al. demonstrated a lower insulin response to oral glucose overload, suggesting decreased secretion or increased insulin sensitivity.^25^

Another study demonstrated a decreased insulin response to venous glucose overload, suggesting a hypoinsulinemic state in patients with CD due to pancreatic denervation or injury.^24^In 2012, Silva et al. published an insulin tolerance test study . They observed that patients with CD had a lower hemodynamic response to increased blood pressure and heart rate than the control group. This diminished response suggests that the sympathetic denervation found in CD impairs the response to insulin action.^29^Nagajyothi et al., in a study using an animal model, demonstrated reduced insulin secretion in the acute phase of CD that persists until the chronic phase, probably as a result of pancreatic inflammation and pancreatic ß cell dysfunction.^30^

Santos et al. (1999) reported a higher frequency of diabetes mellitus and hyperglycemia in women with CHD compared with control groups (digestive form and indeterminate form). As an explanation, they suggested excessive sympathetic activity due to parasympathetic denervation and/or hypoinsulinemia due to functional and anatomical impairment of the pancreas.^31^

It is worth remembering that patients with HF of other etiologies have elevated insulin levels and insulin resistance.^32 , 33^Therefore, the reduction in insulin levels found in our study may indicate the presence of a protective factor against insulin resistance or factors that interfere with insulin secretion and/or action in CD.

There are few studies evaluating insulin levels in infectious diseases, with one showing increased insulin resistance^34^and others showing pancreatic involvement with loss of pancreatic beta cells and reduced *in situ* insulin expression.^35 , 36^Therefore, the findings of our study may be due to peculiar changes associated with CD, such as autonomic dysfunction interfering with pancreatic insulin secretion (pancreatic denervation). In addition, the acute phase of CD may cause morphological and functional changes in the pancreas, leading to chronic involvement,^23 ,37^which could explain the presence of reduced insulin levels even after treatment. Further studies evaluating specific markers of pancreatic injury, as well as indices of insulin resistance, may better explain these mechanisms.

This study was the first to demonstrate that the decrease in insulin levels occurs from the pre-treatment acute phase and becomes even more important in the post-treatment phase of ACD. The possible mechanisms for this decrease can be discussed as compensatory mechanisms related to the development of CHD, the presence of autonomic dysfunction, exacerbated inflammatory activity and/or pancreatic involvement.

The absence of correlations, in this group of patients, between assessment variables of the ANS and cardiac function, makes it necessary to study in more detail the correlations of metabolic markers with inflammatory cytokines and markers of pancreatic involvement. In addition, further studies are needed to assess insulin resistance to better detail the pathophysiological mechanisms and clinical repercussions.

The limitation of this study was the reduced number of patients, mainly due to the difficulty of access to the Amazon region. For this reason, the sample size was not calculated, and the convenience sample was used. The evaluation of a larger number of patients in the future may allow a better understanding of the mechanisms and clinical repercussions of the alterations found in this study.

## Conclusion

Insulin levels were lower in the ACD group both before and after the treatment when compared to the control group and was lower in the post-treatment phase when compared to the pre-treatment phase. Adipocytokine, leptin and adiponectin levels showed no differences between groups. There was no significant correlation between adipocytokine and insulin levels with cardiac function and the ANS parameters.
